# Deep Learning‐Based Monitoring of European Hamster Activity

**DOI:** 10.1002/ece3.74267

**Published:** 2026-09-04

**Authors:** Marie Wittekind, Paul W. Dierkes, Sebastian Schneider

**Affiliations:** ^1^ Bioscience Education and Zoo Biology Goethe University Frankfurt Frankfurt am Main Germany

**Keywords:** activity, AI, camera trap, *Cricetus cricetus*, deep learning, European hamster

## Abstract

In order to ensure the effective conservation of the critically endangered European hamster ( *Cricetus cricetus* ), there is a necessity for the implementation of targeted conservation measures and reliable monitoring methods. This study explores the potential of employing Deep Learning (DL) to assist with camera trap monitoring for the purpose of tracking hamster activity. To this end, an object detection model (YOLO) was trained to efficiently analyze large volumes of video data from summer 2023 with high reliability. The model achieved a weighted average F1‐score of 0.93 and an accuracy of 0.93 for the detection of European hamsters, effectively differentiating them from other species. A comparison between DL‐based and human evaluations confirmed that DL can reliably depict hamster activity patterns. The findings of this study suggest that European hamsters exhibit peak activity levels at dusk, with the highest peak in activity occurring around sunset. In contrast, activity levels were lowest around midday. Autocorrelation analysis revealed a biphasic activity pattern, with a secondary peak occurring approximately before sunrise. This study underscores the potential of employing DL for long‐term conservation efforts and its applicability in assessing the success of reintroduction programs.

## Graphical abstract

We evaluated the use of a deep learning‐based object detection model (YOLO) to automate camera trap video analysis for monitoring the activity of the critically endangered European hamster ( *Cricetus cricetus* ). The AI‐based approach achieved high agreement with human assessments (F1‐score and accuracy of 0.93) and reliably detected temporal activity patterns. Application to long‐term field data revealed a biphasic activity pattern with peak activity at dusk and a secondary peak before sunrise.

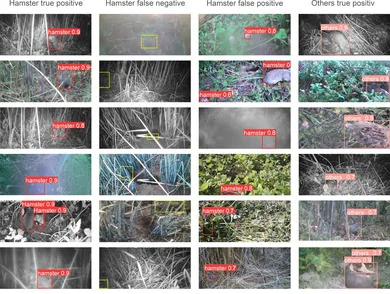

## 1 Introduction

The European hamster ( *Cricetus cricetus* ), once regarded as a pest in many parts of Europe, has now been classified as “critically endangered” by the IUCN (2019). This alarming shift is also evident in Germany, where the species was once widespread but has now experienced a significant decline, with populations in some areas completely disappearing by influences like climate change, changes in agriculture, habitat fragmentation, and genetic impoverishment (Nechay 2000; Reiners et al. 2011; Surov et al. 2016; Tissier et al. 2016). To counter this decline, various conservation measures are being implemented in agricultural landscapes. These efforts aim to enhance habitat conditions and genetic diversity. Important strategies can include managing land to benefit hamsters by planting specific crops and delaying harvests to provide shelter and food (La Haye et al. 2014, 2020). Reintroduction programs have been initiated, with hamsters bred in captivity and released into suitable habitats (La Haye et al. 2017; Descamps and De Vocht 2025). However, the success of these reintroduction projects hinges on multiple factors (La Haye 2010; Robert et al. 2015; Crees et al. 2016).

For long‐term success, sustainable financial resources are essential to support conservation efforts (La Haye 2010). Equally important is the involvement of key stakeholders, for example, nature conservation organizations and farmers. Their active participation in conservation and reintroduction efforts is crucial, as they manage much of the habitat where these projects are taking place (La Haye 2010; Crees et al. 2016). Beyond stakeholder engagement, threats to the hamster population need to be identified and mitigated and the restoration and protection of habitats must be a priority (La Haye 2010; Crees et al. 2016). Scientific monitoring plays a critical role here, as it provides the data needed to assess the effectiveness of conservation efforts and adapt strategies as necessary (Butchart et al. 2010; La Haye 2010). Understanding the activity patterns of the European hamster is important for improving its long‐term conservation. Studies have shown that hamsters are primarily crepuscular, with peak activity occurring in the evening and early morning, influenced by environmental parameters (Monecke and Wollnik 2005; Kaim et al. 2013; Hędrzak et al. 2018). Factors such as crop cover, day length, human influence, and predator presence play important roles in shaping these behavioral patterns (Wollnik et al. 1991; Monecke and Wollnik 2005; Kaim et al. 2013).

Human activity also impacts hamster behavior. For example, hamsters living near urban environments exhibit a later and more intense morning activity peak compared to those in rural settings. This could be due to artificial light, altered food availability, or adapted behavior in response to predators (Kaim et al. 2013; Hędrzak et al. 2018). Such differences highlight the need to consider local conditions when planning conservation and reintroduction efforts.

By gaining a better understanding of hamster activity, conservationists can optimize reintroduction processes and monitoring efforts. Predator presence can be monitored and possible changes in hamster behavior can serve as early warning signs for habitat disturbances. In the conservation of European hamsters, established methods such as radio‐tracking individuals and mapping burrows have been widely used (Kayser et al. 2003; Kletty et al. 2019; La Haye et al. 2020; Descamps and De Vocht 2025; Thürkow et al. 2025). In recent years, wildlife cameras have become a valuable tool for observing animals in their natural habitat, helping to monitor population density, behavior and predator interactions (McCallum 2013; Trolliet et al. 2014). These cameras have traditionally been employed for more controlled research settings, such as studying predator presence, litter size, or juvenile emergence (Albert et al. 2014; Tissier et al. 2018; Kletty et al. 2019; Fleitz et al. 2024; Descamps and De Vocht 2025). To our knowledge there is no peer reviewed publication that uses camera traps to track the activities of European hamsters.

Camera traps are often used as supplementary tools in field research, but they hold considerable potential for broader, long‐term monitoring efforts. However, they do have limitations, such as the need for manual image review, which can be labor‐intensive when large amounts of data are collected. One of the most promising advancements in species monitoring is the use of Deep Learning (DL) to analyze the vast amounts of data generated by camera traps. Deep Learning (DL), a specialized subfield of Artificial Intelligence (AI), has proven particularly effective in this context, as it can automate the identification of specific species or individuals from image data, saving time and reducing the need for manual review (Santangeli et al. 2022; Vélez et al. 2023). In this way, AI‐based analysis can support nature conservation measures, for example, by identifying animal species or through real‐time alert systems (Dertien et al. 2023; Vélez et al. 2023; Hitchcock et al. 2026). Various methods can be used, such as recording audio, camera trap footage, drone imagery, and GPS data that can be automatically evaluated (Onyebuchi et al. 2024; Kitzes et al. 2026). However, there are several challenges associated with using DL for European hamsters camera trap monitoring. First, distinguishing hamsters from other similar species, such as mice, can be difficult, especially when animals are captured at odd angles, under different lighting conditions, or only parts of the animals are visible. Another challenge arises from densely growing crops or complex backgrounds, which can obscure hamsters, making them difficult to detect, particularly when they are farther from the camera and appear as small figures in the image. This can lead to errors in identification or missed observations, impacting the accuracy of the data (Gomez Villa et al. 2017; Norouzzadeh et al. 2018; Zhang et al. 2020). Despite these hurdles, Deep Learning methods have advanced significantly and can closely match human accuracy in analyzing image data, particularly when well‐trained datasets are available (Norouzzadeh et al. 2018; Green et al. 2020; Fennell et al. 2022). Moreover, DL presents a cost‐effective solution, with many tools available at low or no cost beyond the initial setup (Norouzzadeh et al. 2018; Vélez et al. 2023). As DL continues to evolve, it has the potential to revolutionize species monitoring by increasing efficiency and accuracy to gain information about social behavior or hazards (Mallick 2025). Overcoming challenges like species misidentification or obscured views will be critical to maximizing the potential of this technology in conservation efforts.

While DL‐assisted monitoring has become a standard tool in various ecosystems (Tuia et al. 2022; Fergus, Chalmers, Longmore, and Wich 2024; Rahmati 2024), its application for the European hamster remains unexplored, with no peer‐reviewed studies currently utilizing camera traps to track the activity of this species. The primary challenge lies in the immense volume of data generated during long‐term monitoring, which makes manual review labor‐intensive and often impractical. In this study, we utilize a validated DL approach (YOLOv8n) as a functional tool to process over 133,000 video sequences. Our objective is to demonstrate that a high‐performance automated workflow can reliably substitute manual analysis to provide high‐resolution insights into the activity patterns of reintroduced hamster populations. By validating the DL‐derived data against human expert assessments, we aim to establish a robust baseline of crepuscular and nocturnal behavior, ultimately providing a scalable method to evaluate the success of reintroduction programs and refine conservation strategies for this critically endangered species.

## 2 Methods

We use three test sites dedicated to the reintroduction and protection of European hamsters. Two reintroduction areas, each two hectares in size, are located in the district of Gießen in Germany, specifically in Langgöns and Pohlheim. Another reintroduction area is situated in Frankfurt Bergen‐Enkheim in Germany and covers 2.4 ha. All areas are completely enclosed with electrified fencing to protect against predators. The diverse plant offerings, including alternating strips of flowering plants, legumes, and cereals, provide food and shelter (Figure S1). Late harvesting of the fields in September and October ensures that the European hamsters can find ample food and protection from predators for an extended period.

European hamsters were released into the wild at 1 year of age, except for one 2‐year‐old male released in Pohlheim. Release numbers differed among sites. In 2023, 14 hamsters were released in Bergen‐Enkheim (7 females, 7 males), 21 in Langgöns (14 females, 7 males), and 24 in Pohlheim (14 females, 10 males). In Langgöns, an additional 12 hamsters had already been released in the previous year and reproduced successfully. Due to mortality and variations in reproductive success across the different areas, the number of hamsters varied throughout the season in all areas.

For camera monitoring, wildlife cameras were installed in front of burrows suspected to be inhabited by European hamsters from June 2023 until the end of the season in September 2023. Camera placement decisions were based on evidence such as feces at the burrow entrance or other accompanying investigations like regular recaptures of the hamsters. A total of 29 cameras and four different camera models were used for the monitoring (5 Maginon WK 1, 8 Victure HC300, 6 Stealth Cam STC‐G42NG, 10 Wosports Trail Camera G600). The distribution of camera models was random, ensuring that each model was represented on every site. The number of cameras per site depended on the number of released European hamsters. In Bergen‐Enkheim, where the fewest European hamsters were released, a maximum of seven cameras was set up. In Langgöns and Pohlheim, a maximum of 11 cameras were in operation. The position of cameras was changed if no or only very sporadic activity was detected at a hamster burrow.

The optimal positioning and setting of the cameras were determined at the beginning of the fieldwork. They were placed at a distance of at least 80 cm on a wooden stake to ensure that the entrance to the burrow was visible. Any plant parts obstructing the view were removed from the camera view. The low height and the chosen perspective also ensure that people captured by the camera cannot be recognized. This ensures that the research was conducted in a socially responsible manner and that privacy was not violated. Ten‐second‐long videos were recorded and a low sensor sensitivity was set on the cameras. The cameras were checked approximately once a week. To address the primary objective of analyzing hamster activity patterns across a large‐scale dataset, we developed an automated analysis pipeline. This approach was chosen to ensure time‐efficient and standardized processing of the data, which would have been unfeasible through manual review alone.

### 2.1 Object Detection

The object detection model employed in this study was YOLOv8n (nano), developed by Ultralytics. Python version 3.10.9 was used as the programming language. The YOLOv8n architecture was selected for this study due to its optimal balance between computational efficiency and predictive performance, which was essential for processing the extensive dataset of 133,163 videos. As the “nano” variant of the YOLOv8 family, it is specifically designed for high‐speed inference without compromising the accuracy required for ecological monitoring. Videos were opened using OpenCV. Training data images were extracted from videos at a rate of 3 frames per second (fps). To minimize temporal autocorrelation and prevent overfitting to specific movement sequences, only non‐consecutive frames from distinct video segments were selected for the final training set. This sampling strategy ensured that the model learned to identify morphological features across a wide range of lighting and environmental conditions rather than memorizing specific temporal sequences. The model was trained for 100 epochs with a batch size of 16 and an image size of 640 × 640 pixels. To ensure a stable and efficient learning process, we used an initial learning rate of 0.01 and a momentum of 0.937, which helps the model maintain a consistent direction during optimization. Additionally, a weight decay of 0.0005 was applied as a regularization technique to prevent overfitting, ensuring that the model generalizes well to new, unseen data rather than just “memorizing” the training images. These settings, combined with data augmentation, including horizontal flipping, HSV‐space adjustments, scaling and translation, were critical for maintaining robust detection performance across the varying light, distances and vegetation conditions encountered in the agricultural fields.

The training set comprised two classes: “hamster” and “others,” with the latter encompassing all other species recorded, such as mice, owls, raccoons, and cats. Image counts per class are provided in Table 1. Since image frequency was determined by field recordings, the classes are naturally unbalanced. To ensure the model captured maximum seasonal and environmental variability, training frames were randomly sampled throughout the entire field season from June to September. While strict separation between datasets was prioritized, a post hoc audit identified a marginal overlap of 73 training frames (< 0.2% of the test volume) originating from videos also included in the test set. This overlap is considered statistically negligible, particularly as our pipeline's requirement for temporal consistency (2% occurrence threshold per video) ensures that detections rely on generalization across the entire sequence rather than the recognition of individual frames. The model was evaluated against an independent test set of 37,362 human‐labeled videos, ranging from 2 to 10 s in length, representing typical field recordings. These videos, which had been labeled by humans, were then also classified by the trained model to assess how well the model performs on unseen data. The trained model predicted each video in the test dataset frame‐by‐frame. This approach was taken to ensure that temporal relationships and class distributions across the video were effectively captured. To enable comparison with a human evaluator, the ratings for all frames in a video were aggregated and the video as a whole was assigned to a class. Consequently, hamster activity is represented by the total number of videos in which a hamster was detected. The model returned information about detected objects, including their class IDs, confidence scores and bounding box coordinates. Only predictions with confidence scores above a predefined threshold of 0.65 were considered for further analysis. The confidence threshold of 0.65 was selected by evaluating model performance across a range of potential values (0.6, 0.65, and 0.7). As summarized in Table S1. The 0.65 threshold yielded the lowest median deviation (6.50%) between the DL model and human assessments, effectively balancing the rates of false positives and false negatives. Detected class occurrences were aggregated over all frames. Classes with a relative occurrence below 2% of the total frames per video were discarded to minimize errors. The most frequently occurring class was designated as the primary label for the video. This prediction pipeline enabled robust detection of hamsters in videos, taking into account both the predictions for individual frames and their temporal distribution over the entire video. By incorporating thresholds for both confidence and occurrence, the method ensured that the assigned labels were both meaningful and reliable.

**TABLE 1:** Number of images used to train the model (train and validation) in total and the number of labeled boxes per class. In addition, the number of videos used to test the trained model (Test‐set) and the number of found classes in the videos are shown.

Train			Validation			Test‐set		
Hamster	Others	Images	Hamster	Others	Images	Hamster	Others	Videos
1708	777	2409	474	176	634	7808	2297	37,362

To calculate the daily activity of the hamsters, the number of discrete videos (2–10 s duration) with positive hamster detections was aggregated into 30‐min interval bins based on their respective recording timestamps. Therefore 133,163 videos, produced between June and September 2023, were analyzed via the trained object detection model. If monitoring measures took place in the reintroduction areas, these days and the following day were removed from the data set to avoid possible influences on the natural animals' behavior. To assess periodicity in hamster activity, we computed the autocorrelation of the activity data using MATLAB (version R2023a). The autocorrelation was calculated using the xcorr function with normalization to obtain correlation coefficients between −1 and 1. Peaks in the autocorrelation function indicate recurring activity phases, with the time lag corresponding to the interval between peaks.

## 3 Results

**FIGURE 1:**
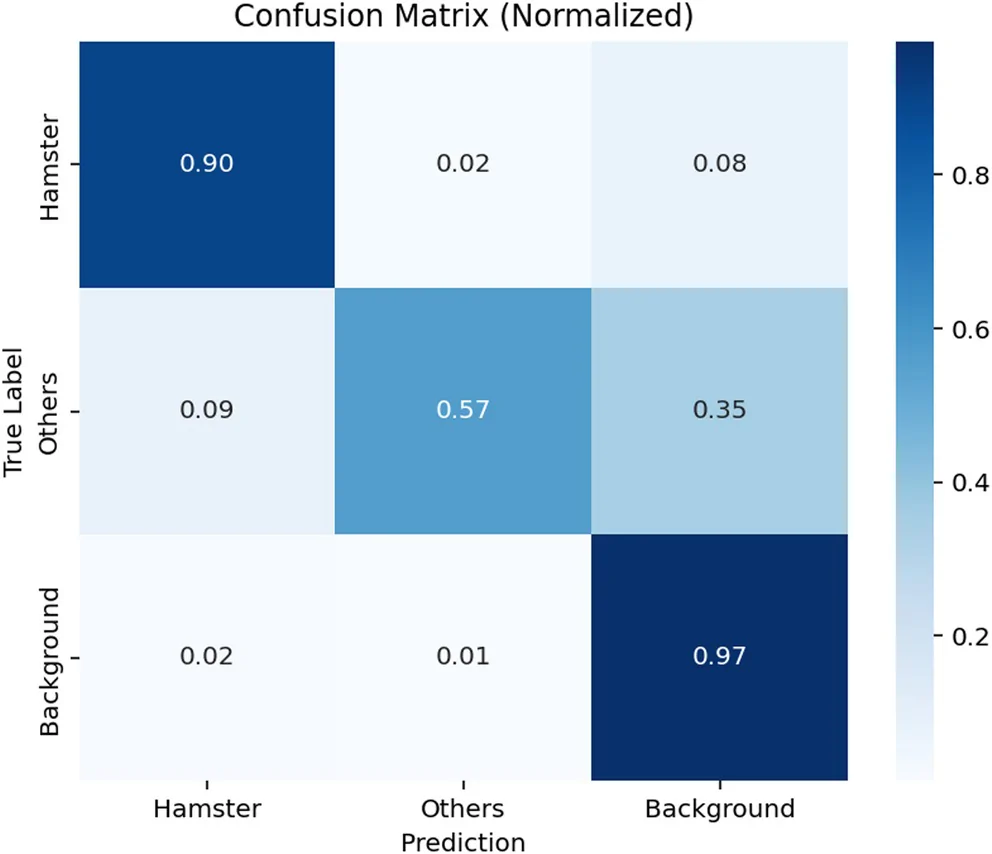
Normalized confusion matrix showing the classification performance of the YOLOv8n model. The matrix compares the DL predictions (horizontal axis) with the human expert evaluations (vertical axis, “True Label”) for a test set of 37,362 videos. Three classes are represented: European hamsters, other animals (“others”), and empty recordings (“background”). The values are normalized, where the diagonal cells represent the percentage of correctly identified instances for each class.

In the test data, hamsters were identified by humans in 7808 videos. The confusion matrix (Figure 1) shows the prediction of the DL model compared to human observations (“True Label”). The model was able to reliably identify 90% of the videos in which hamsters appeared, 8% remained unidentified and 2% were mistaken for another animal. Of the 2297 videos in which other animals such as rats, mice, or rabbits were seen, the trained model incorrectly identified a hamster in 9% of cases. Overall, the DL model achieved an accuracy score of 0.93 [95% CI: 0.927–0.933] and a weighted average F1 score of 0.93 (Table 2).

**TABLE 2:** Class‐wise and aggregated performance metrics of the model using the test set, including precision, recall, and F1‐score. The results are reported for the classes Hamster, Others, and Background, along with macro and weighted averages.

	Precision	Recall	F1‐score	Support
Hamster	0.92	0.90	0.91	7808
Others	0.74	0.57	0.64	2297
Background	0.95	0.97	0.96	27,257
Macro Avg	0.87	0.81	0.84	37,362
Weighted Avg	0.93	0.93	0.93	37,362
Accuracy	0.93			37,362

**FIGURE 2:**
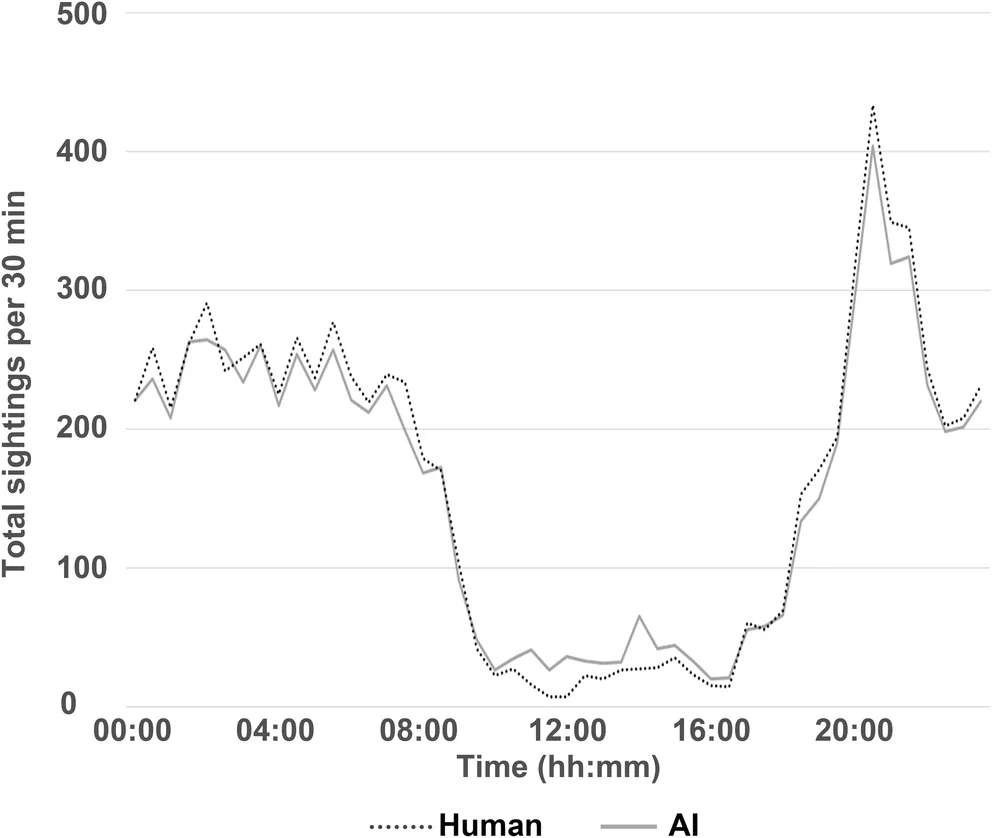
Comparison of human and DL rated European hamster activity. The total number of videos in which hamsters were detected per 30‐min interval is shown.

The analyzed test‐set videos were used to estimate the activity of the hamsters throughout the day. Figure 2 shows the activity of the animals over the course of the day, assessed once by humans and once by DL. The two activity curves are nearly continuously parallel and differ only slightly. The median deviation of the DL model compared to humans is 6.5% (Table S1). The deviations during the day, when the activity is overestimated, are greater than during the active phases at night, when it is slightly underestimated.

**FIGURE 3:**
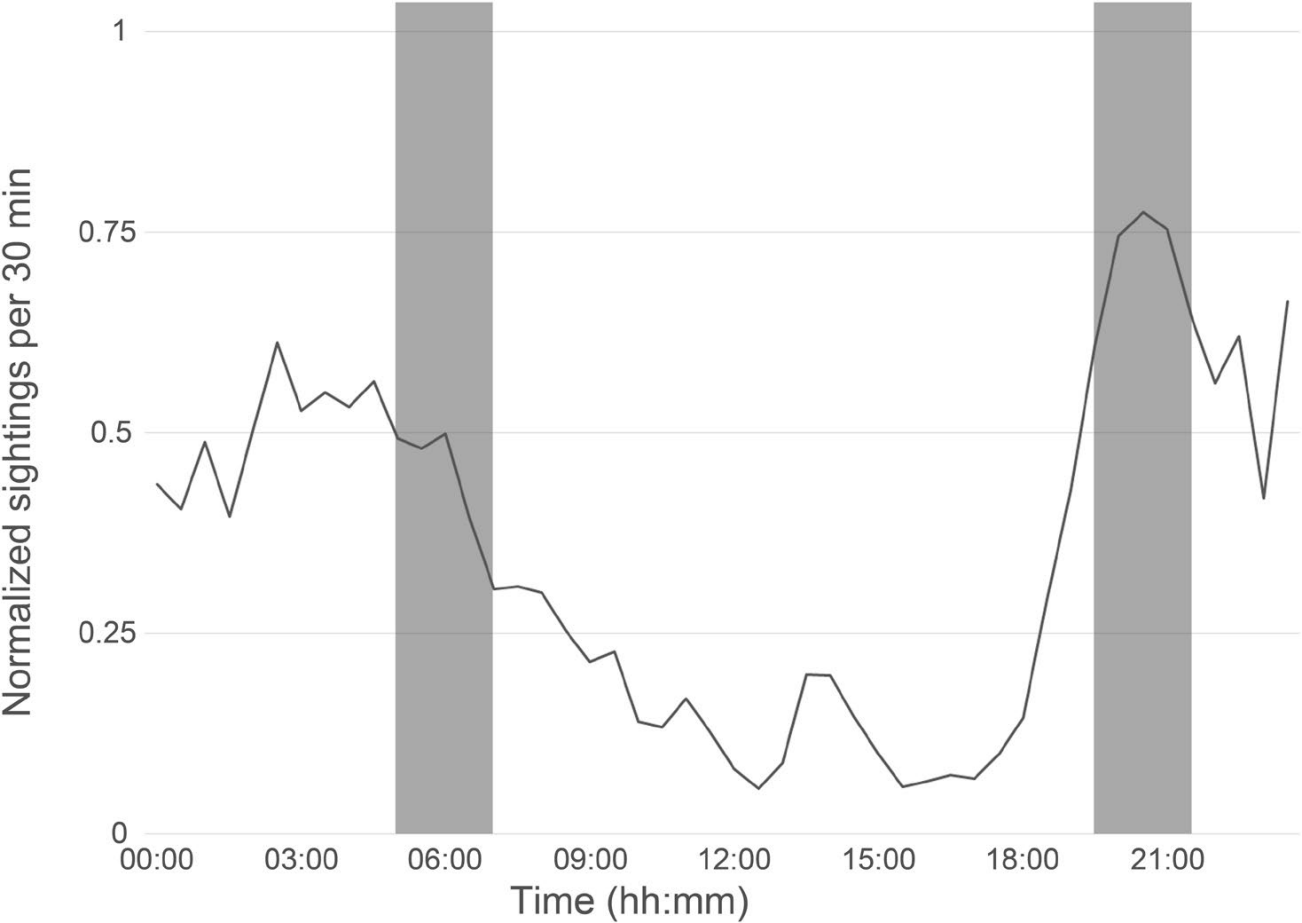
Above‐ground activity of European hamsters between June and September per 30‐min interval. The gray marks show the period of sunrise and sunset. Activity data were normalized by dividing each 30‐min interval by the monthly maximum and then averaging the results across months.

To estimate the activity pattern of the European hamster during the summer month, 133,163 videos were analyzed by DL (Figure 3). Plotting the activity of European hamsters showed that they are primarily crepuscular and nocturnal. The highest peak of activity is reached at sunset at around 8:30 p.m. Following this, activity levels decrease but remain high until sunrise. After sunrise, activity levels drop and stay low throughout the day (Figure 3).

**FIGURE 4:**
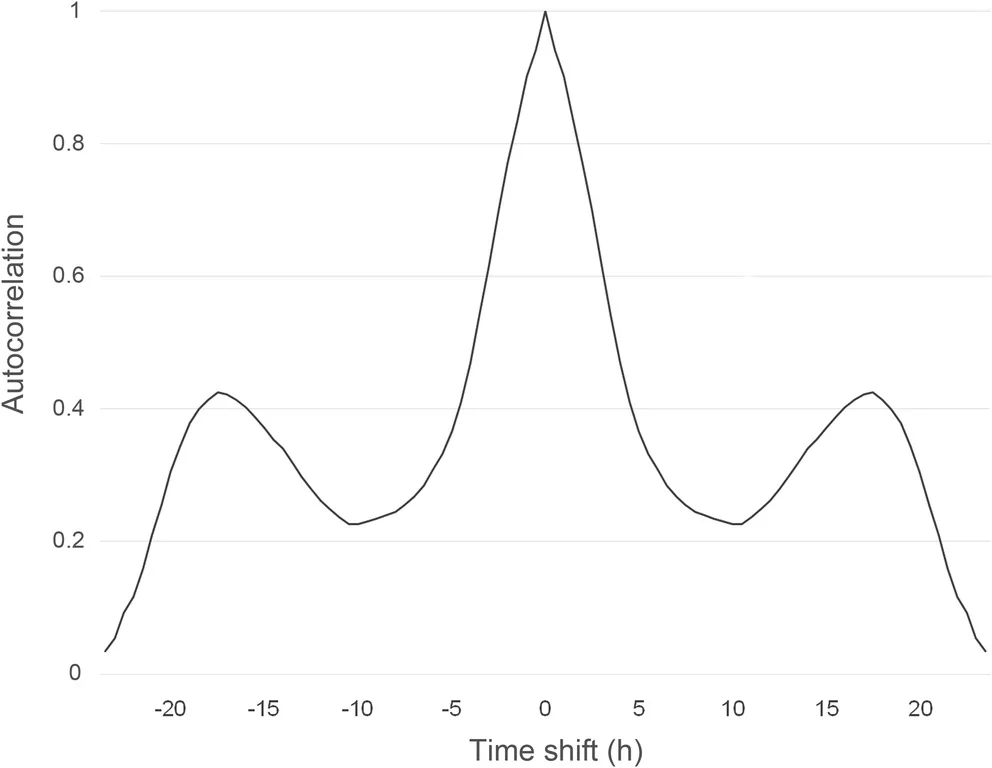
Autocorrelation of hamster activity data. The primary peak at zero represents perfect self‐correlation, while the secondary peak at 17.5 and −17.5 h indicates a recurring activity phase, confirming a biphasic activity pattern.

Autocorrelation of the data confirms a biphasic activity of the hamsters (Figure 4). The secondary peaks at 17.5 and −17.5 in the autocorrelation reflect the recurring activity pattern. This lag corresponds to the interval between the first peak at sunset (around 8.30 p.m.) and the second activity phase at around 3.00 a.m., as shown in Figure 3.

To further investigate the nature of the false positive rate, we conducted a qualitative spot check of 200 randomly selected videos where the DL model predicted a hamster but humans did not. This review revealed that in 16 of these 200 cases (8%), the DL model correctly identified hamsters that mostly were partially obscured or difficult to discern and had been overlooked during the initial human evaluation. In total, 22 human errors were identified within this subset (including misidentifications and missed detections). This suggests that the reported DL error rate is partially inflated by human oversights, confirming the model's high sensitivity even in complex visual scenarios.

## 4 Discussion

Testing the trained object detection model has demonstrated that it is a functional tool for determining the above‐ground activity patterns of European hamsters. By employing this DL technology, we provide the methodological foundation for obtaining more detailed and continuous insights into their life. Particularly in long‐term monitoring, where extensive data must be analyzed, DL can significantly alleviate human workload. For instance, manually evaluating the 133,163 videos collected in this study would require an estimated 370 h of labor (assuming a conservative average of 10 s per video for review and data entry). By automating this process, the DL pipeline not only reduces the risk of human fatigue and subsequent erroneous assessments but also enables the processing of large‐scale datasets that would otherwise be logistically and financially prohibitive for manual review. Beyond efficiency, the use of automated camera traps also addresses methodological biases. Previous studies often investigated European hamster activity in human presence (Kaim et al. 2013; Hędrzak et al. 2018), which limits observation time and may influence animal behavior. While this paper focuses on validating the automated workflow and establishing a behavioral baseline, it opens the door for future research to holistically investigate how environmental variables interact with these patterns.

This becomes evident in the activity data, which shows a biphasic pattern. While the biphasic activity pattern itself is consistent with earlier findings (12, 13), our study provides a novel level of resolution and objectivity. By utilizing an automated DL‐pipeline to analyze over 133,000 videos, we were able to record these patterns without human interference, a known limitation of previous observational studies. Furthermore, the high consistency between our results and established biological knowledge serves as a critical validation that DL‐assisted camera‐trap monitoring is a reliable and scalable tool for long‐term hamster conservation, which has not been demonstrated in peer‐reviewed literature to date. The greatest peak in activity is reached around sunset (8:30 p.m.) and the second approximately at 3 a.m. The influence of environmental factors such as disturbances was not investigated, but may play a role as other studies have shown (Kaim et al. 2013; Hędrzak et al. 2018). Characteristic for European hamsters is the significant reduction in activity during midday that occurs. This behavior may be attributed to adaptations to environmental conditions, such as predator avoidance (Kaim et al. 2013; Hędrzak et al. 2018).

### 4.1 Limitations and Challenges

Camera trap footage cannot reflect the all‐encompassing activity of the hamsters. The cameras were placed at the entrances to the hamster burrows and therefore mainly record animals entering and leaving the burrow. Individuals that leave the burrow quickly and spend most of their time outside the field of view of the camera are therefore underrepresented compared to individuals that spend more time in the field of view of the camera. Due to the relatively high number of cameras (29) and the long recording period of 4 months, these effects are likely to be less relevant when it comes to assessing the overall above‐ground activity of the animals.

The use of camera traps during the monitoring is susceptible to environmental influences. For example, camera traps can fall over if the ground is soft, hamsters can tamper with cameras, and cold temperatures can lead to accelerated battery drain. Therefore, it is crucial to carefully set up the experimental design and conduct regular checks. This allows us to generate substantial data, necessitating adequate storage capacity. To conserve storage space, images could be captured instead of videos. This approach extends the operational time of the cameras and saves storage space.

Regarding DL‐based analysis, accuracy varies depending on image content. For instance, the color composition within the image can influence classification quality (Tan et al. 2022). Shadows cast during daylight may have contributed to misclassification, which could potentially be addressed through additional training (Norouzzadeh et al. 2018; Schneider et al. 2020). This is consistent with the results shown in Figure 2, which showed that DL model misjudges more frequently at midday, where there is a considerably greater deviation from the human assessment at this time. In addition, delays between animal detection and actual image or video capture can lead to distortion of hamster activity. A significant technical challenge is the trigger delay of camera traps; hamsters moving rapidly through the field of view may only be partially captured at the frame edges, which hinders DL detection. While human observers can often identify moving animals from partial shapes in a video sequence, the DL model, relying on the classification of individual frames, is more susceptible to these incomplete representations. However, our validation using the 37,362‐video test set demonstrates that the impact of these hardware‐related false negatives is minimal (8%) and the overall activity patterns remain highly consistent with human observations, showing a median deviation of only 6.5%. Thus, while camera delays represent a known limitation, they do not significantly distort the ecological findings of this study. For future applications, utilizing cameras with shorter trigger intervals or transitioning to image‐based capture could further mitigate these detection gaps. Nevertheless, the object detection performance is indeed impressive, considering that the image quality was often affected by fogged lenses, dense vegetation or blurred images (Figure S2). At the same time, occasional misclassifications still occur. Random checks also revealed that plants were among the false positive hamster detections (Figure S2). Other studies that use DL to analyze camera trap data have reported frequent misclassifications caused by moving vegetation, shadow artifacts and complex backgrounds, highlighting the need for training schemes that cover environmental variability (e.g., shadow, thermally heterogeneous scenes) and improve reliability via dynamic training sets (Maile et al. 2023). Habitat‐specific error rates (e.g., open grasslands causing more “empty” misclassifications) further underline this challenge (Egna et al. 2020). These parallels suggest that model refinement through more balanced training datasets and class restructuring is a general requirement across taxa, supported by work showing that two‐phase training, where models are first trained on majority classes and then fine‐tuned on minority classes, can mitigate imbalance (Malik et al. 2021) and that domain expert ensembles, which combine multiple specialized sub‐models instead of a single classifier, improve recognition in long‐tailed camera‐trap datasets (Park et al. 2022).

Our findings reflect these challenges at the level of dataset design. We categorized the detected animals into two classes: “hamster” and “others.” The class “others” was used to group all non‐hamster animals found in the training data, including a wide range of species such as birds and various mammals. This high variability within the class poses a challenge, as the heterogeneity in size and shape of some animals can increase the risk of misclassification, where hamsters could be misclassified as others or vice versa. To improve the performance of the model, future efforts could split the species into multiple classes and use a larger number of images for training to improve class discrimination.

In the analysis of discrepancies between identifications made by DL and human observers, it is important to acknowledge that human error cannot be entirely excluded. Despite careful review processes, there is a possibility that hamsters may be overlooked or misclassified as another species by human observers, while the DL model correctly identifies them. While managing the extensive dataset of 133,163 videos from 29 individual cameras required complex data integration, we ensured data integrity through regular cross‐checks. The high level of agreement between DL and human observers further confirms that the data handling and classification processes yielded robust results. While human error is often assumed in large‐scale monitoring, our spot check of 200 suspected false positive hamster videos quantified this impact, finding 16 human errors where the DL model had performed correctly. This confirms that the human “gold standard” is subject to fatigue or oversight, particularly in dense vegetation. Consequently, DL does not merely replicate human performance but acts as a robust secondary verification tool that can enhance overall data integrity.

Finally, it should be noted that, even though DL yielded satisfactory results, its training data were generated within the study areas and are, therefore, to some extent optimized for the data subsequently analyzed. Ultimately, testing the DL model on new reintroduction sites will serve to validate its quality.

### 4.2 Future Work

Given the reliability of the trained Yolo model demonstrated in this study, its application offers various benefits for future monitoring projects. DL can streamline workflows by identifying periods of low above‐ground activity for disturbance‐prone tasks and optimizing resource allocation. Moreover, analyzing activity patterns with DL can provide insights into behavioral responses to environmental changes, predator presence, or human disturbance. Over time, such data could inform predictive habitat models. By identifying patterns in hamster activity relative to environmental factors (e.g., temperature, vegetation cover, or human activity), researchers can predict optimal habitats and identify potential disturbance factors. Additionally, integrating age‐related activity differences into future analyses could deepen understanding of population dynamics, reproductive behavior and stress responses. For instance, Prendergast et al. (2012) demonstrated that activity rhythms in female Syrian hamsters vary substantially across reproductive stages such as gestation and lactation (Prendergast et al. 2012). While no peer‐reviewed studies explicitly contrast urban versus rural hamster populations, integrating habitat‐related variables, such as agricultural, peri‐urban, or urban landscapes, into camera‐trap analyses could yield ecologically richer and more informative models.

The potential of DL extends beyond European hamsters, enabling multi‐species monitoring through the automated detection and classification of various wildlife species captured in camera trap images. By training models to recognize multiple species simultaneously, researchers can gather data on species composition, abundance, and activity patterns within an ecosystem. This broader application of DL facilitates the study of species interactions, such as predator–prey dynamics or competition, and provides a more comprehensive understanding of ecosystem health. Additionally, DL‐based data can identify changes in biodiversity over time, offering valuable insights into the impacts of environmental changes, habitat loss, or climate change on wildlife populations. These ecosystem‐wide insights can inform conservation strategies aimed at protecting not only target species but also the entire ecological community they inhabit (Tuia et al. 2022; Fergus, Chalmers, Longmore, and Wich 2024; Nel et al. 2024; Rahmati 2024). Similar multi‐species DL frameworks have already been applied in other ecosystems: for example, Deep Learning analyses of large mammal communities in African savanna and Asian dry forests provided robust assessments of species richness, occupancy, and activity patterns, highlighting the importance of data quality and size in ecological inference (Bevan et al. 2026). New ways are emerging to identify birds based on their calls and to monitor migratory bird populations (Nel et al. 2024; Yadav 2025). The skillful application of DL systems can contribute to the prevention of human‐wildlife conflict (Ojija et al. 2025). By analyzing data and satellite imagery, the quality—or even the disappearance—of habitats can be detected and assessed, enabling the rapid implementation of measures for their protection (Albuquerque et al. 2022; Casas et al. 2023).

Several global platforms demonstrate the applicability of multi‐species DL frameworks. Vélez et al. (2023) compared popular platforms, including Conservation AI, MegaDetector, MLWIC2, and Wildlife Insights, and highlighted their effectiveness in monitoring species distribution, activity, and biodiversity across diverse environments (Vélez et al. 2023). Fergus et al. (2024) further demonstrate how a global platform (Conservation AI) has been deployed successfully in regions such as Europe, North America, Africa, and Southeast Asia, using CNNs and Transformer models for species detection and behavioral monitoring (Fergus, Chalmers, Longmore, and Wich 2024). A subsequent study found that integrating YOLOv10‐X with vision‐language models provided an enriched ecological context. This integration not only reported species presence but also vegetation type and temporal information, thereby enhancing ecological inference (Fergus, Chalmers, Matthews, et al. 2024).

The high reliability achieved in this study (F1 = 0.93) is consistent with broader findings where large‐scale CNN models have proven robust under challenging conditions. For example, Tabak et al. (2019) demonstrated that models trained on over 3 million images can capture species with near‐perfect accuracy (98%) and generalize well even to out‐of‐distribution data (Tabak et al. 2019). Similarly, Pantazis et al. (2024) confirmed that ecological metrics such as species richness, occupancy and activity derived from DL models remain reliable, even under conditions of data noise and limited sample sizes (Bevan et al. 2026).

Taken together, these advances suggest that applying multi‐species DL approaches to European agricultural landscapes could significantly enhance conservation monitoring for European hamsters and other co‐occurring species, providing more comprehensive insights into ecosystem dynamics and informing effective conservation strategies.

## 5 Conclusion

This study demonstrates the potential of a trained Deep Learning object detection model for reliably detecting European hamsters in field camera data, achieving an accuracy score of 0.93 and a weighted average F1 score of 0.93. The model identified 90% of hamster videos correctly in a test data set of long‐term recordings. Despite some challenges, such as higher misclassification rates during daylight hours and the variability of the “others” class, the model produced activity patterns closely aligned with those assessed by human observers, with a median deviation of 6.5%. The activity data showed that the European hamster has a biphasic, mainly crepuscular and nocturnal activity, with the highest activity at sunset and the lowest activity during midday. These findings highlight the potential for Deep Learning to complement traditional methods in long‐term monitoring programs, offering a scalable solution for studying activity patterns and supporting conservation efforts. However, it should be noted that the current model is optimized for a binary classification task (“hamster” vs. “others”) within this specific study system. While highly effective for broad temporal monitoring, future refinements with more diverse multi‐class training data would be required to further reduce false positives from non‐hamster species in varying ecological settings.

## Author Contributions

**Marie Wittekind:** conceptualization (equal), data curation (equal), formal analysis (equal), investigation (equal), methodology (equal), validation (equal), visualization (equal), writing – original draft (equal), writing – review and editing (equal). **Paul W. Dierkes:** funding acquisition (equal), project administration (equal), resources (equal), supervision (equal), writing – original draft (supporting), writing – review and editing (supporting). **Sebastian Schneider:** conceptualization (equal), data curation (equal), formal analysis (equal), software (lead), supervision (equal), validation (equal), visualization (equal), writing – original draft (equal), writing – review and editing (equal).

## Funding

This study was supported by the Opel‐Zoo Foundation Professorship in Zoo Biology from von Opel Hessische Zoostiftung.

## Conflicts of Interest

The authors declare no conflicts of interest.

## Supporting information


**Table S1:** The table presents the discrepancy between DL predictions and human evaluations across three distinct threshold values for the DL model's prediction probability. The deviation is indicated once in the total number of videos per 30‐min interval and once in a percentage deviation per 30‐min interval.

**Figure S1:** Spatial overview and agricultural composition of the three hamster reintroduction sites. The maps show the distribution of specific crop strips at (A) Langgöns, (B) Pohlheim, and (C) Bergen‐Enkheim. The diverse planting strategy, featuring alternating strips of flower mixes, cereals, alfalfa, sunflowers, field beans, and wheat, is designed to provide continuous food resources and cover from predators throughout the field season. Red outlines indicate the designated reintroduction areas within the agricultural landscape. Map data provided by the Feldhamsterschutz working group of the Hessische Gesellschaft für Ornithologie und Naturschutz (HGON), based on satellite imagery from Google Maps. OpenStreetMap (https://www.openstreetmap.de) was used for the large‐scale overview map.

**Figure S2:** Examples of detection outcomes from the trained object detection model. The figure is organized into four columns showing representative results for different detection categories: (1) true positives for “hamster” detections (red bounding boxes with label and confidence score), (2) false negatives where hamsters are present but not detected (yellow bounding boxes), (3) false positives where objects were incorrectly classified as hamsters, (4) true positives for “others” class.

## Data Availability

The trained YOLOv8n model, the Python scripts for training and prediction, and the underlying evaluation datasets are available in the Goethe University Data Repository (https://doi.org/10.25716/gude.1azk‐2b01). These datasets include the comparative data for the AI versus human performance assessment (supporting the results in Figure 2) as well as the long‐term activity data derived from the analysis of 133,163 videos collected between June and September 2023.
