# Supplementary material for: Deep Learning‐Based Monitoring of European Hamster Activity
Source: Ecol Evol. 2026 Sep 4;16(9):e74267. doi: 10.1002/ece3.74267 (PMC13542996; doi:10.1002/ece3.74267)
Supplement: Supplementary file 1 [file ECE3-16-e74267-s001.docx]

Supporting Information

Table S 1: The table presents the discrepancy between DL predictions and human evaluations across three distinct threshold values for the DL model’s prediction probability. The deviation is indicated once in the total number of videos per 30-minute interval and once in a percentage deviation per 30-minute interval.

| Threshold | 0.6 | 0.65 | 0.7 |
| --- | --- | --- | --- |
| median deviation (total numbers) | 12.5 | 10.5 | 11 |
| median deviation  (%) | 6.61 | 6.50 | 7.21 |
